# Dental Abstracts

**Published:** 1854-01

**Authors:** 


### Dental Abstracts.

In the October number of the American Journal of Dental Science, for 1853, we have an article on filling teeth, when complicated with inflammation of the pulp. By C. Spencer Bate, Esq., Plymouth, England.

To show the practice of some of our brethren in England we give two or three extracts from this article.

Jan'y 30.—Miss S., of London, came on a visit to her relations in Swansea, (where I resided and practiced for twelve years), for her health, her constitution generally being debilitated. She called upon me relative to two central incisor teeth of the upper jaw, both of which were decayed, the right tooth loose, the gum inflamed and swollen, and an abscess developed beneath the lip. The whole hadbeen for many days, and still continued, acutely pairful. Upon my commencing to prepare the cavity for plugging, she expressed her doubt of its utility, because three times within two years had they been alreedy done in the metropolis, but with no beneficial result. The filling soon became loose, and after a time came out, leaving the hole larger than before. She, therefore, would have preferred the entire removal of the tooth and an artificial substitute.

However, I proceeded with the operation, removed much of the decay but did not expose the pulp, though the decayed portion reached the central cavity of the tooth. I ordered a leech to the gum, an aperient and rest, and dismissed her after having filled the tooth with *gutta percha stopping.*

What I call *gutta percha stopping* is a mixture I make myself, of plaster of paris and gutta percha, mixed before the fire until the latter will take no more without crumbling. This I find a useful temporary stopping, and after it has been in the mouth a few minutes presents no excessive contrast with the tooth.

February 10.—On this day my patient again visited me. I found the tooth firmer, with less swelling. The leech had been misapplied and took only on the lip; the tumor got worse, and a second leech was more successful, removing the pain and bringing down the swelling. I opened the abscess on the gum, filled permanently the left incisor, which was not complicated in the inflammatory action which had attacked the right.

March 5.—Found the tooth firm and free from pain, abscess gone, a slight redness with scarcely perceptible swelling remained in its place, but which my patient informed me had not been so much the day before as now. The temporary stopping was not well in. She had suffered no pain since I last saw her, but the tooth had lost its color, looking a little gray, the pulp was dead and some dark tissue remained, which was removed by boring into the pulp cavity, then plugged the tooth, adopting a plan recommended in one of the American journals, of putting in a piece of white paper to prevent the gold from being seen through the transparent enamel. I separated the two incisors with a file, and ordered a leech to the spot where the abscess had previously been, more as a precaution than from any immediate necessity.

September 16.—Filled to-day some teeth for the above patient's uncle, to whom she had been on a visit while in Swansea. He informed me that she had been perfectly comfortable since I saw her last, and had now returned home.

In this case, we are informed that an abscess had "developed" itself "beneath the lip," the carious portion was partially removed, "but did not expose the pulp, though the decayed portion reached the central cavity of the tooth." A "leech was ordered to the gum, an aperient and rest." The tooth was then filled with "gutta percha" stopping, and the patient dismissed.

A natural enquiry here arises ; was there any danger of exposing the pulp after the formation of an abscess? Would not the escape of the matter from the abscess, by the use of a lancet, prove more beneficial than the leech? Why fill the tooth under such circumstances with any substance?

We should at once open the abscess ; in two or three days remove the decay, and then for a few days introduce into the nerve cavity of the tooth a solution of Chloride of Zinc or Creosote and Tannin, and as soon as soreness passed off fill the fang and tooth.

In the second case we have an ingenious method to "defend the living dentine from the rude contact of the gold," and the persistence in a course of treatment which, although tedious and painful, yet, in the end, appears to have saved the tooth. We should like to know if the nerve is still alive.

Miss J., upon hearing that I was about to leave Swansea, asked me to examine her mouth. Among other things which I thought necessary to be done, I found a tooth which I had plugged many years before, the gold having shown signs of wearing from mastication, I therefore removed it. I found the cavity healthy, except a little softening of dentine near the surface where a fragment of enamel had broken away. Sometime since, she had been conscious of some uneasiness about the tooth. Removed the incipient caries and manipulated a little, chiefly with a view of improving the shape of the cavity, in order to give durability to the plug. While using a fine broach for the purpose, and pressing with some force, it slipped from its place and broke through the thin wall of dentine into the pulp cavity. The immediate result of such clumsiness on my part was pain of rather a severe character, but which fortunately was not continuous; a small quantity of blood oozed through the perforation. Over this I placed a thin solution of gutta percha dissolved in chloroform, and then pressed in the gold and completed the stopping.

The next morning, to my great discomfort, the lady reported that she had suffered much pain and could not drink cold water, and warm tea also gave her pain. I begged for two days trial, as pain often continues for a short time after the operation, the result of irritation from cutting dentie in a healthy state.

At the termination of the period agreed upon, she stated that the pain was less acute, but still more than she could possibly bear. Consequently, I removed the plug, reduced a gnawing pain by the topical application of Chloroform, and dismissed her with a little cotton in the tooth.

March 10, a week after. The tooth was so far restored that she feared to have it filled again. But the tooth, though easy, I found, upon examination after the removal of the plug, to be exquisitely sensitive to the slightest touch of the instrument. In order to defend the living dentine from the rude contact of the gold, I cut a thin section of dentine from the tooth of a walrus, and to render it pliant, I dipped it for a few minutes in hydrochloric acid, after washing it well to free it from all trace of acid, I cut it to the proper form, laid it at the bottom of the cavity and pressed the gold upon it. The commencement of the operation was painful, but was ultimately successful, so that when tested with cold water, it remained free from pain.

On the 24th, that is thirteen days after, my patient called and reported that for some days the tooth was easy, but suddenly (to use her expression) "something in the tooth seemed to give way," since which it had been very painful, but the night before the ache appeared to settle in the second under molar, which had been filled long previously, but which, upon being carefully examined, was found to have a small carious spot which burrowed into the tooth and became complicated with the cavity already plugged. The latter I tore out and re-filled the tooth, which bore the opeiation well, though severe pain was caused at first by the application of tra. camph. to the denuded cavity; but no inconvenience was felt upon pressing in the gold—which was leaf. Presuming that the pain felt was caused by this last tooth, I sent my patient away without meddling with the upper molar, the tooth in question.

On the 26th, I found the last mentioned tooth (upper molar) still severely annoying her. I again removed the filling, upon which, we were both cognizant of a slight but offensive effluvia. The gold which came in contact with the ivory-plate was slightly darkened in color, which convinced me that, however careful I might have been in washing the plate, still some acid must have remained to the disadvantage of the tooth and risk of the operation, although the ivory plate was so perfectly and firmly attached to the bottom of the cavity, that I could not immediately remove it.

I now refilled the tooth temporarily with gutta percha stopping, which remained in without any deterioration until the 9th of September, when I found the small communication through the floor of the cavity closed up by dentine. I now filled the tooth without any false bottom to the cavity. It bore the operation with impunity, and remained well when I last heard of it, and I have no doubt but that it will continue to be permanently successful.

We admire the candor and perseverance of Mr. Bate, and although differing much in practice, yet we are indebted to him for some good ideas. We give in conclusion his plan to "relieve an inflamed pulp:"

The plan which I have generally adopted, in order to relieve the pain consequent upon an inflamed pulp, is first to observe if the tooth be tender on being brought into contact with those in the opposite jaw; if this be the case, without any distinct looseness in the tooth itself, the result of an inflammatory action in the alveolar membranes. The tooth should be isolated from its neighbor, by the use of a file which should distinctly preclude any contact so as to interfere with the natural articulation of the tooth itself when the two jaws meet. In such cases, the use of the file to an exceedingly painful tooth, is less disagreeable than the same application to a more healthy one, and relief is apparent immediately upon the decided separation of the tooth from the other. Upon this being completed, I return to the cavity and proceed to remove as much as possible of the superincumbent gangrenous osseous tissue, by taking off the pressure from the confined and swollen pulp, to allow it to have free scope for its increased dimensions. This is easily done by means of sharp excavators; but should the tissue be hard, the pulp being exposed only by an almost impervious opening, I generally use a rose drill hollowed in the center, or a broach similarly constructed so that I can cut all round the chamber without injuring the pulp. Having proceeded sufficiently deep, the bottom of the cavity should, if possible, be removed in one piece, care being taken that no splinter or spiculæ of dentine be left in contact with the irritated pulp; for I believe this is not unfrequently the cause of an unsuccessful treatment.

Having giving room to the pulp to relieve itself by swelling, I endeavor to reduce any irritation which may continue, by the application of either saturated tra-camphoræ, chloroform or comphorated chloroform, to both the exposed pulp and soft tissue of the mouth near the seat of pain.

### *Osseous Union of the Teeth.*

In the October number of the Journal, Mr. M. K. Bridgeman reports a case, the union of the right central and right lateral incisors of the lower jaw of the permanent set.

The same number of the Journal contains an article on the physical condition of the teeth, the causes which determine it, and, as a consequence, the difference in the liability of these organs to decay, by A. A. Blandy, M. D., D. D. S. This article is to be continued, and when completed we may make some extracts; our present limits forbid this now.

The same number of the Journal contains a very interesting case of "spontaneous inflammation of the alveolo-dental membrane," by C. A. Harris, M. D., D. D. S. After having extracted a lower molar of one side, and in a few weeks its mate on the other side, which had become excessively painful, and had resisted constitutional and local treatment, he remarks that:

Seven or eight weeks after the last operation, she visited me again. Two other teeth, an upper molar and a lower bicuspid, had become the seat of constant, gnawing pain. Both of these teeth were slightly affected with caries, but the structural alteration had penetrated but a short distance into the dentine, and could have had no agency in the production of the pain, which, as in the two former cases, was evidently the result of periodontitis, and that not caused by any other source of local irritation than the mere presence of the teeth, but dependent upon great preternatural irritability of the periosteum, arising from some peculiar cachectic habit of body, or state of the general health. Entertaining this view of the case, and not wishing to interfere with the general treatment which seemed evidently to be indicated, I advised her to have leeches applied to the gums of the affected teeth, and to place herself under the care of her physician, to whom, at her request, I addressed a note, expressing my opinion with regard to the cause of the pain from which she was suffering. As she resided in the country, some twelve or fifteen miles from Baltimore, I had great difficulty in persuading her to return with the aching teeth in her mouth, but yielding to my solicitations, she finally consented to do so. She returned immediately, sent for her physician, and was at once put under medical treatment, which was perseveringly continued for about seven weeks. During this time, aperients, tonics, such as quinine and the various preparations of iron, counter irritants and narcotics were prescribed; but the pain continued without any mitigation, and in the meantime, extended to two of her other teeth. It had become so agonizing, that she was unable to obtain any sleep at night, except when under the influence of large doses of morphia, and despairing of relief, she again visited the city, firmly resolved to have the four aching teeth removed. Her suffering was now so great, that I no longer refused to perform the operation. The roots of these teeth presented he same appearance as those of the two first.

Miss T. left Baltimore, the day after the operation, comparatively free from pain; but the sockets remained sore, and at times, slightly painful for several weeks.

About three months after the removal of the last teeth, another began to ache, and in about three weeks, the pain having assumed such a degree of severity as to render its longer endurance almost insupportable, she came to the city and had the tooth extracted. The loss of this procured a few weeks freedom from pain; but in a short time, another was seized, and was ultlmately removed. In this way, tooth after tooth was attacked and extracted, until at the expiration of about eighteen or twenty months, all of the molars and bicuspids, except one, of both jaws, were removed.

*Socket Plate.*—The same number of the Jonrnal contains also an article on mounting single porcelain teeth on a socket plate, by C. A. Harris, M. D., D. D. S., giving Dr. Hullihen's method. This is illustrated with some good engravings, yet the plan is so similar to that given in the 3d No. of Vol. VI, Dental Register, by Dr. J. Taft, of Xenia, Ohio, for forming sockets for gum teeth, that a special description is not necessary. We prefer the plaster, as used by Dr. Taft, for getting the impression to work by in fitting the rim which forms the socket.

The Oct. No. of the Dental News-Letter has several interesting articles, which we should be glad to notice more in full than our present limits will permit. The first is an address by Prof. E. Townsend, before the American Society of Dental Surgeons, at its last annual meeting. The subject, Dental Patents. The Doctor has taken a full and comprehensive view of the subject, and although we may differ in some respects from him, yet we cheerfully give him credit for the able manner in which he has handled the subject. After discussing the principles involved in patent right and copy right, the Doctor closes his address as follows, by remarking that the positions which I (that is he) take, are to the following effect:

The natural right that any man has in any discovery he may make in art or science, is not a *patent* right. The former, at the utmost, only entitles him to conceal and use, as best he may, his invention for his own benefit. If he wants a better hold and profit of it than this, the patent law offers him a compromise bid for it, which amounts to this:—

"If you will at once give up your secret, the community will give up their right to discover it for themselves for a given time, and surrender all other claim, moral, social and natural, which they may have to such beneficial truths as God has so stamped with freedom, that they can not be held by any man for his private profit a moment after they are ushered into being." This, says the law, shall be the bargain for a term of fourteen years. Take it or refuse it, and trust to your natural ingenuity to conceal, as well as to discover, the thing.

There, I conceive, stands the matter of patent-rights, to whatever subject applied. For my life I can see no other merit in the claims it is used to protect. In fact, we know that few really great discoverers have ever been able to make anything for themselves out of their special privileges. Hucksters and peddlers, as usual, fatten on the spoils which genius has won for the world. The *natural* right, I again assert, is the right to keep the secret, not to sell it to all the world, and compel them to *buy* or do without it.

Now, how does such a right stand in professional life? Suppose I am called in consultation with a physician, surgeon, or brother dentist, can I, without violating every implied pledge of such consultation, keep my secret, if I have a helpful one, from my associates, and from our patient's service? The answer is a plain one, and the inference is just as plain, that the natural right which would induce, excuse or warrant a violation of duty and honor in professional life, ceases to be a right within the sphere of that profession. That is all that I affirm against dental patents. They are unprofessional, and, for all the reasons which ought to govern us, should be put under our interdict. Again, I wish it to be understood that I am addressing my argument specially to that foundation in natural justice, which has been claimed for dental patents. If the occasion served, and I were treating the matter in a wider view, I should consider it and the advocacy to be encountered, in a different way, and it seems to me not without success. There results, also, this other consequence from the ground taken here, that if dental patents are unprofessional, I may, if I please, buy a right, if any offers, which duty to my patients requires me to employ, but I may *not* sell such privilege of use. When I purchase a patent or thing, the vender has put himself out of the profession, by refusing, or despising its obligations, and with my views of the matter, whenever I, by any act, word or hint, sign or other form of agreement to the thing, make myself the agent, instrument, or accessory to the act of vending the same, I put myself out. This is not the same thing, be it observed, as saying anything inconsistent with the courtesy and respect I otherwise owe to any one of my brethren involved in the manner that I would myself condemn, but it *is* saying and meant to say, that all dental associations, colleges, and practitioners who have the responsibilities of the profession upon them, and enjoy any of its benefits and honors, should unhesitatingly put dental patents under ban, and treat them as they properly do all other monopolies in the industrial and liberal arts, as things outside of the pale of the fraternity.

The view here taken as the object of the patent law, is perhaps different from that usually entertained, which is that it is designed to encourage and stimulate discovery and improvement, and as an incitement offering her protection. In discussing the nature of a patent right, the Doctor remarks, "It is to be kept in mind, moreover, that patent right properly applies only to those combined results of *knowledge* and *skill* which produce physical, material, or mechanical results."

This we believe is true. That is, a principle of science can not be patented. The electric fluid can not be prohibited from general use by a patent, but only a certain mechanical mode of applying or using it; and as we regard it, it is this mechanical production which the patent law protects for the benefit of its author, just as the literary production is protected for the benefit of its author.

The results of this state of things on our profession is, however, a different affair, and as we have before remarked, on another occasion, "The medical profession regarding the cure of disease as of the first consequence, that in assuming the duties of the profession, they impose on themselves a moral obligation which shall consult first, the preservation of life—since it has become a settled principle in medical ethics, that all knowledge essential to this great object, should be free; that all remedies for the cure of disease should become the property of the profession."

Now it is the moral obligation which renders the patenting of anything which is necessary to the cure of disease, unprofessional. Every one knows that occasionally, mechanical contrivances are necessary in the treatment of disease, and when such are patented by the profession, the patentee disregards this moral obligation and renders himself amenable to those laws which govern the medical profession. We regard Dental Surgery, in every sense of the word, as a department of this profession.

We regard the individual who deals in specific nostrums and secret remedies, as a Quack. He attempts to make his fortune off the credulity of the public. The patentee places himself boldly before that public, having disclosed his secret and under cover of the law, tries to make *his* fortune, and was it not for that paramount obligation, he may owe the public, as a professional man, which he disregards for his own ends, he would not be considered censurable.

If, then, the patentee is also a Quack, he is a lawful one—having made a "bargain" with the law-making power to act in this capacity for a certain period of years. Let this, however, be as it may, (and we do not intend to quarrel with the profession about it), the question comes up, shall we discard the patented article, because it is such? If duty to our patients requires that we employ a patented article, do we throw ourselves out of the profession by its purchase? We regard our duty to our patients as first and paramount, and hence differ with Prof. Townsend in this particular. But we do not intend to discuss this question, particularly at this time.

The same No. of the News Letter contains an Inaugural Thesis, by J. D. White, M. D., D. D. S. The subject of this thesis is, "The treatment of exposed dental pulp, preparatory to the operation of filling." After speaking of the various modes recommended by different authors, he notices the use of Arsenic, and gives the following as a mode of preparation and application. It will be recollect this thesis was written in 1843–4:

*Arsenious Acid, Kreasote, and Morphia.*—This is, perhaps, the best form of using arsenic that has yet been devised. I have not seen it spoken of by any authors; there is no difference in the rapidity with which it unites with the pulp when the kreasote only is properly combined with it; but the morphia will exert its narcotic influence, and lessen the pain, an effect which we can not ascribe to kreasote; the kreasote can not be regarded in any other light than a mere vehicle.

℞.—Arsenious acid, gr. xxx.
Morphiæ sulphas, gr. xx.
Kreasote, Q. S.
Misce.

Put the arsenious acid and kreasote in a glazed mortar, and grind them till the arsenic becomes impalpable, (adding kreasote to keep the mass of about the consistency of cream); then add the sulphate of morphia, and mix it well, still adding kreasote; it will dissolve in the paste. Prepared in this way, the arsenic is in a better condition to unite speedily with the pulp than the mere dry powder of arsenic, on account of the kreasote holding a large quantity of it in solution, and it becomes more minutely divided. Great care must be taken to cleanse out the external cavity of the tooth, so as to place the paste in immediate contact with the pulp. A pledget of cotton about the size of a small pin's head, steeped in the paste is sufficient. If the pulp bleed when the cavity is cleansed, we must wait till the bleeding subsides before we apply the paste; the cavity may then be filled with cotton, and left in from ten to twenty hours. If it be in the tooth of a young patient, the bone will, perhaps, absorb a sufficient quantity of the arsenious acid to inflame the alveolo-dental membranes, and of course it should be removed in such cases in a shorter time than it could be left with safety in the case of an older patient, or a dense and opaque tooth.

In commenting on the effects of the remedy, the Doctor remarks:

The pain was most severe, and of greatest duration in patients of a strong nervo-sanguine temperament; but even in those cases, if the pulp had been subject to frequent attacks of inflammation, it rarely gave pain when the paste was applied. Again, patients of a scrofulous diathesis rarely suffered pain, whether the pulp had been previously subject to inflammation or not. The paste should not be allowed to spread over the walls of the external cavity, expecially when applied in the teeth of young patients. It should never be applied in the teeth when there is on account of the age of the patient, reason to believe that the roots are not fully developed. If the pulp be destroyed, the further formation of the tooth will be arrested. Intense inflammation will be produced in the external membranes and jaw, by destroying the pulp outside of the incompletely formed roots.

In the same number of the News Letter, we have an article on the "Warping of plates in soldering," by W. E. Ide, M. D., Columbus, Ohio." After referring to the expansion and contraction which takes place in the operation of soldering and cooling on the "*investments* of plaster," &c., and on the metals, he gives the following process:

For whole sets of teeth I procure copper plate as thick as is consistent, with a reasonable degree of pliability, and cut into strips about an inch and a half wide, and long enough to make a circle somewhat larger than the circumference of a set of teeth. This strip is bent into a ring, and the ends rivited together. I next pierce this copper ring with two rows of holes, one row near the upper border, and the other a half inch below the first, and the holes about half an inch apart. I then take well-annealed copper wire, as large as can be readily wrought with the fingers and pliers, and weave this through the holes in the ring on the inner side, leaving the wire in short but even lopes. This leaves a net-work of strong wire on the inside of the ring. The size of the wire which I use is about No. 14. I then cut into the lower edge of the ring to near the lower row of holes, at very short intervals, quite around its circumference. The sections of plate thus cut are turned in toward the center of the ring. When the teeth are arranged on the plate in wax, I bend the ring to near the shape of the piece containing the teeth, and put the latter in the former. The net-work of wire should nearly or quite touch every tooth—the space between the teeth and copper plate not being more than half an inch. I then prepare a thin mixture of two-thirds plaster and one-third clean sand, and pour in to fill the space between the teeth and copper ring. After the mixture hardens I invert the piece, and pour upon the palatine surface of the plate a small quantity of plaster and sand, in which there are at least five parts of sand to one of plaster. This last is merely to keep up the evenness of the heat in the process of soldering. It will be noticed that the quantity of plaster and sand in this process is unusually small. This I consider important for two reasons: In the first place, it diminishes the contractile force of the plaster. Second, it enables the operator to solder with a less amount of heat, and also to keep the temperature more even. This last fact renders the danger of cracking the teeth positively *less* than in the usual way. The usual practice is to use a large amount of plaster and sand with a view of preventing the teeth from cracking. This, I am convinced, is all a mistake. As little should be used as will keep them firmly in place. It will be noticed that the plaster and sand first put in the ring is perforated in all parts with the wire net-work. It also does not occupy the whole circumference of the ring, but only that which is bounded by the teeth. Now, as the piece is gradually heated, the expansion of the copper ring and net-work being greater than that of the gold plate, and the plaster mixture being carried along with the ring, all contractile force upon the gold is effectually prevented. The last mixture of plaster and sand that is used on the palatine surface of the plate, has such a preponderance of sand that there is no contractile tendency from that source. I always raise the piece to a red heat at first in a charcoal furnace, and in soldering I take good care to direct the blaze occasionally upon the outside of the ring, so that it may not contract while the plate is expanding. After the teeth are soldered on I restore the whole to the furnace, and cool gradually, taking care the circumference is kept at a higher heat than the part which contains the plate. If the process here described is strictly adhered to, and the gold plate not too much alloyed, I think springing never need be feared. According to my experience, a plate never springs under any ordinary heat, when heated by itself for the purpose of annealing. This is to my mind a fact confirmatory of my theory, that the cause of the warping is always found in some force acting upon the circumference of the plate. The fact that when such force is prevented, the plate *does not warp*, is a demonstration to me satisfactory. Let those who doubt try it.

